# Associations between family structure and young people’s physical activity and screen time behaviors

**DOI:** 10.1186/s12889-019-6740-2

**Published:** 2019-04-25

**Authors:** Amund Langøy, Otto R. F. Smith, Bente Wold, Oddrun Samdal, Ellen M. Haug

**Affiliations:** 1 NLA University College, Bergen, Pb 74 Sandviken, 5812 Bergen, Norway; 2 Department of Health Promotion, Norwegian Institute of Public Health, Bergen, Zander Kaaesgate 7, 5015 Bergen, Norway; 3 Department of Health Promotion and Development, University of Bergen, Christiesgate 13, 5020 Bergen, Norway

**Keywords:** Family structure, Young people - physical activity, Sport participation, Screen time behaviors

## Abstract

**Background:**

Identifying factors that can influence young peoples’ physical activity and sedentary behaviors is important for the development of effective interventions. The family structure in which children grow up may be one such factor. As the prevalence of single parent and reconstituted families have increased substantially over the last decades, the objective of this study was to examine whether these family structures are differentially associated with young people’s MVPA, participation in organized sports and screen-time activities (screen-based passive entertainment, gaming, other screen-based activities) as compared to traditional nuclear families.

**Methods:**

The data stem from the 2013/2014 “Health Behaviour in School- aged Children (HBSC) study”*.* A large Norwegian sample of 11–16 years old students (*n* = 4509) participated. Cluster-adjusted regression models were estimated using full information maximum likelihood with robust standard errors (MLR).

**Results:**

After adjusting for covariates, living with a single parent was negatively associated with days/week with 60 min MVPA (b = −.39, 95%CI: −.58, −.20), and positively associated with hours/weekday of total screen time (b = .50, 95%CI: .08, .93). Young people living with a single parent were also more likely to report no participation in organized sports (OR = 1.40, 95%CI: 1.09, 1.79). Living in a reconstituted family was negatively associated with days/week with 60 min MVPA (b = −.31, 95%CI: −.53, −.08), and positively associated with hours/weekday of total screen time (b = .85, 95%CI: .37, 1.33). For all outcomes, the interaction effects of family structure with sex, and with having siblings were not statistically significant. For material affluence, a significant interaction effect was found for participation in organized sports (χ^2^ [4] =13.9, *p* = .008). Those living in a reconstituted family with low or high material affluence had an increased risk for not participating in organized sports whereas those with medium material affluence did not.

**Conclusion:**

This study suggests that living with a single parent or in reconstituted families was unfavorably associated with physical activity, sport participation and screen-based behaviors among Norwegian youth. The findings indicate that family structure could be an important factor to take into account in the development and testing of interventions. More in-depth research is needed to identify the mechanisms involved.

## Background

Physical activity is associated with physiological [1, 2], psychological and social [3] health benefits for children and youth. A disconcerting trend with lower numbers fulfilling the recommendations of 60 min/day of moderate to vigorous physical activity (MVPA) is observed, with an overall age-related decline during adolescent years [4]. Simultaneously, young people’s time spent on sedentary activities is increasing [4–6] and such activities may dominate their lives today [7]. Computer use for gaming and non-gaming purposes is sharply increasing and seems to counterweight the observed decrease in TV viewing [4]. Sedentary time and screen-based activities are negatively associated with young people’s physical health, other adverse health behaviors and socio-cognitive outcomes [8, 9]. Several countries have developed guidelines, often recommending that the after-school period should be limited to two hours per day with screen-based activities [10–13].

According to the behavioral epidemiology framework, factors that correlate with young peoples’ physical activity and sedentary time need to be identified to inform the development of effective intervention strategies [14]. Based on a comprehensive review, Biddle et al. (2011) found parental influences to be the key issue regarding social and cultural correlates of physical activity in children. In particular, the existing literature suggests that parental support, either directly or indirectly affects children’s physical activity level in three important ways; encouragement, involvement and facilitation, including financial resources [15, 16]. There is a paucity of research when it comes to the relationship between parental influences and young people’s sedentary behavior, and so far, only parental TV viewing appears to be a consistent correlate [8]. Nonetheless, results from interventions designed to reduce sedentary behavior suggest that the involvement of family and parents was an important ingredient for success [17].

The extent to which parental influences exert a positive or negative effect on young children’s level of physical activity and sedentary behaviors, may also depend on the family structure in which children grow up. Children and youth are increasingly living in various types of family structures. In the EU the numbers of divorces [18], and the number of children being born of single parents, are growing [19]. Similar patterns are present in countries outside the EU, such as Australia [20], Canada [21] and China [22]. Research on family structure has increased dramatically the past two decades, drawing a complicated picture of family conditions and processes that are associated with healthy child development. In general, the literatures suggests that living with single parents or in reconstituted families are less favorable in both the cognitive and emotional-behavioral domains [23], also in the context of the generous Nordic welfare states [24]. Simplified, the two primary mechanisms that might account for differences among families are time and money [24]. A higher likelihood of reduced economic resources in single-parent and reconstituted households has been documented [24–26]. Studies also suggest that children may receive less parental time and attention in both single-parent families and stepfamilies [24, 27].

The studies that have examined whether family structure is associated with young peoples’ physical activity level [28–30], participation in sport [31–35], and screen time use [20, 36, 37], have so far resulted in inconclusive findings [20, 38, 39]. One major limitation is that family structure have almost exclusively been defined as simply single- or dual-parent, thereby ignoring the potential differences between traditional dual-parent families and reconstituted families that include a step-parent or a parent’s partner [16].

Correlates for physical activity and sedentary behaviors are likely to be behavior specific. Organized sports is one of the most popular activities among young people [40, 41] and can contribute to a significant amount of young people’s total physical activity [42, 43]. Few studies have identified correlates of sedentary activities with an explicit attention to the after-school period [44], and those that exist have mainly focused on TV viewing [8]. The objective of this paper was therefore to examine the association between various family structures (living with both parents, a single parent or in a reconstituted family) and young people’s MVPA, organized sport participation and different types of after-school screen time use, including a reference to the existing guidelines.

## Methods

### Participants

The data was collected as a part of the 2013/2014 survey of the “Health Behaviour in School- aged Children (HBSC) study: A WHO collaborative national study”. This cross-sectional survey had school class as the primary sampling unit, the classes were chosen from a geographically stratified list, to ensure a nationally representative sample. A sample of 11-, 13-,15- and 16 years old students (*n* = 4509) participated, with a class level response rate of 21% and a student level response rate of 76%. At a school/class level a high work load and frequent requests regarding survey participation was reported as the main reasons for non-response. Absence on the day the survey was conducted, was the most frequent cause of non-response at the student level. The Norwegian Western Regional Ethical Committee (REK) approved the study and the use of passive consent. A detailed information letter was given both in paper form and electronically to parents or custodians for all participants below the age of 16. Those that did not want their child to participate had to sign and return a form to the teacher. Approval of the child’s participation was assumed if the form was not returned. The class teachers administered the survey. Participation was voluntary, and the anonymity as well as the confidentiality of the participants were ensured. More details on the HBSC study procedures can be found elsewhere [45].

#### Measures

*Family structure* was measured with one item “Please answer this first question for the home where you live all or most of the time and tick the people who live there”. The response categories were: Mother, father, stepmother (or father’s partner), stepfather (or mother’s partner) and others (e.g. living with grandparents). The data was coded into four categories (both parents, single parent, reconstituted families and others). Participants in the ‘others’ category (1.8%, *n* = 83) were excluded from the analyses.

*Moderate to vigorous physical activity (MVPA)* was presented to the participants with the following statement: *With physical activity we mean any activity that increases your heart rate and makes you get out of breath some of the time. Physical activity can be done in sports, school activities, playing with friends, or walking to school. Some examples of physical activity are running, biking, dancing, skateboarding, swimming, soccer, skiing/snowboarding. For the next question, please summarize all the time you were physically active for every day.* This description was followed by the item “Over the past 7 days, on how many days were you physically active for a total of at least 60 minutes per day?” There were eight response categories (0 days, 1 day up to 7 days). The item has been proven to have reasonable validity and moderate reliability [46] and acceptable correlation with accelerometer measures [47, 48]. To reflect the recommended 60 min MVPA a day [49], a dichotomized variable with 7 days = 1 and other responses = 0 was computed.

*Organized sport* was measured by the combination of “How often do you usually participate in organized team sports?” and How often do you usually participate in organized individual sports? These items had four categories (Not at all, 2–3 times a month, once a week, twice a week or more). The items were dichotomized with those answering ‘Not at all’ on both items = 1, other combinations = 0. The items have shown acceptable levels of agreement, indicating good reliability [50].

*Screen-based behaviors* were assessed by three items: “How many hours a day, in your free time, do you usually spend watching TV, videos (including YouTube or similar services), DVDs, and other entertainment on a screen?”, “How many hours a day, in your free time, do you usually spend playing games on a computer, games console, tablet (like iPad), smartphone or other electronic device (not including moving or fitness games)?” and “How many hours a day, in your free time, do you usually spend using electronic devices such computers, tablets (like iPad) or smart phones for other purposes, for example, homework, emailing, tweeting, Facebook, chatting, surfing the internet?”. All items had 9 categories (0, 0.5, 1 to more than 7 h). Participants answered these questions separately for weekdays and weekends. The items have had acceptable test-retest correlations [51–54]. A sum score of the three items measuring total hours a day with screen time was computed, and a cutoff at 2 h/day was set to extract an additional dichotomized variable to reflect recommendations.

Previous research has shown associations of young people’s physical activity and screen-based behavior with gender, age, material affluence (SES), having siblings and BMI [31–33, 37, 55–59], and these variables were controlled for.

*Material affluence* was measured by a summary score of The Family Affluence Scale III (FAS-III). The FAS consists of the following six items: Does your family own a car, van or truck? (Responses: no, one, two or more); Do you have your own bedroom for yourself? (No, yes); How many times did you and your family travel out of Norway for a holiday/vacation last year? (Not at all, once, twice, more than twice); How many computers do your family own? (None, one, two, more than two); Does your family have a dishwasher at home? (No, yes); and How many bathrooms (rooms with a bath/shower or both) are in your home? (None, one, two, more than two) [45, 60]. These six items provide a scale that can be easily completed by adolescents. A ridit transformation (conversion to cumulative probabilities) by age and sex was carried out on the sum score of the FAS-III items. The ridit scores were subsequently categorized into three groups with varying levels of relative material affluence: low (lowest 20%), medium (between 20th and 80th percentile) and high (highest 20%) [60].

*Body mass index (BMI)* using the formula kg/m2 was assessed by two items: “How much do you weigh without clothes?” (in kg) and “How tall are you without shoes?” (in cm). The BMI scores were recoded into standardized z-scores based upon the guidelines from Childhood Obesity Working Group of the International Obesity Taskforce [61]. Self-reported BMI tends to underestimate the prevalence of obesity [62–66], and the associations of self-reported BMI with other variables are biased when BMI is used as a categorical variable [67]. The latter bias tends to be much lower when BMI is used as a continuous variable [65, 67].

*Siblings* was measured by two items referring to where the respondent lived all or most of the time: “Please indicate how many brothers and sisters live here (including half, step or foster brothers and sisters)” (How many brothers?, How many sisters?).

### Statistical analyses

SPSS 23.0 was used to calculate descriptive statistics. Mplus 7.4 was used for all other analyses. Due to the clustered sampling design, the “complex” option in Mplus was used to account for dependency between observations from the same school class. Substantial missing data was present for variables; screen-based passive entertainment (17.0%), gaming (19.0%), other screen-based activities (19.7%), parental status (11.4%), BMI (12.9%), participation in organized PA (12.4%), and having siblings (10.9%). Full Information Maximum Likelihood (FIML) with robust standard errors (MLR) was adopted to deal with missing data and non-normality. FIML is valid under the assumption of missing at random, which is generally considered to be a more realistic assumption as compared to missing completely at random. MVPA (8-point scale), screen-based passive entertainment, gaming, other screen-based activities and total screen time (9-point scales) were treated as continuous dependent variables. Although formal normal distribution and homoscedasticity assumptions were not met for these variables, MLR estimation is known to be robust against non-normality and heteroscedasticity. Moreover, comparing ML with MLR estimates (while ignoring clustering) indicated small differences between standard errors, suggesting that the degree of assumption violation was small as well. Binary logistic regression was used for dichotomized versions of MVPA, total screen time, and participation in organized PA. For each dependent variable, the crude association with parental status was calculated first. This was followed by an adjusted analysis accounting for sex, age group, material affluence, having siblings, and BMI. For each continuous outcome and participation in organized PA, the interaction of parental status with sex, material affluence and having siblings was examined by means of a Wald test.

## Results

Table 1 shows the characteristics of the sample. About one quarter was living in a single or a reconstituted family. The large majority (82.8%) did not meet the 60 min MVPA 7 days/week with a mean of 4.1 (+ 2.0 SD) days/week. The proportion not participating in organized sport was 38,2%. The average total screen time was 6,1 (+ 4.3 SD) hours/day, with 83,6% exceeding 2 h/day.

**Table 1:** Baseline characteristics

Variable	Estimate
Female sex, % (n)	52.8 (2381)
Age category, % (n)	
11 yrs. old	30.2 (1353)
13 yrs. old	23.0 (1030)
15 yrs. old	19.4 (869)
16 yrs. old	27.3 (1223)
Material affluence, % (n)	
Low	18.3 (783)
Middle	65.5 (2809)
High	16.2 (696)
Having siblings, % (n)	90.9 (3652)
BMI, mean (SD)	19.7 (3.5)
Family structure, % (n)	
Both parents	74.1 (2962)
Single parent	15.7 (628)
Reconstituted	10.2 (407)
60 min MVPA in days, mean (SD)	4.1 (2.0)
Less than 7 days 60 min MVPA, % (n)	82.8 (3507)
Not participating in organized sport, % (n)	38.2 (1051)
Screen-based passive entertainment (hours/day), mean (SD)	2.1 (1.6)
Gaming in hours/day, mean (SD)	1.7 (1.8)
Other screen-based activities in hours/day, mean (SD)	2.4 (2.0)
Total screen time in hours/day, mean (SD)	6.1 (4.3)
Screen time > 2 h/day, % (n)	83.6 (2935)

In Table 2 the crude associations show that living in a single parent or a reconstituted family structure was negatively related to number of days with 60 min MVPA and increased the odds for less than 7 days/week and the odds for not participating in organized sport. In the adjusted model, living in a single or in a reconstituted family structure remained associated with number of days with 60 min MVPA, while the odds for less than 7 days/week and the odds for not participating in organized sport remained significantly higher for those living with a single parent.

**Table 2:** Crude and adjusted associations of family structure with physical activity^a^

	60 MIN MVPA (days)		Less than 7 days 60 MIN MVPA		Not participating in organized PA	
	b	95% CI	Odds ratio	95% CI	Odds ratio	95% CI
Crude						
Family structure						
Single parent	**-.58**	**(−.78, −.38)**	**1.55**	**(1.19, 2.02)**	**1.66**	**(1.31, 2.10)**
Reconstituted	**-.46**	**(−.69, −.23)**	**1.57**	**(1.13, 2.19)**	**1.33**	**(1.03, 1.72)**
Adjusted						
Female sex	**-.47**	**(−.60, −.34)**	**2.20**	**(1.84, 2.64)**	**1.30**	**(1.05, 1.61)**
Age category						
11 yrs. old	**.89**	**(.57, 1.22)**	**.55**	**(.37, .83)**	–	–
13 yrs. old	**.50**	**(.20, .79)**	.97	(.66, 1.43)	**.32**	**(.24, .43)**
15 yrs. old	.16	(−.11, .44)	.86	(.58, 1.27)	**.64**	**(.49, .83)**
Material affluence						
Low	**-.72**	**(−.93, −.50)**	**1.76**	**(1.31, 2.38)**	**1.77**	**(1.34, 2.34)**
Medium	**-.50**	**(−.67, −.33)**	**1.52**	**(1.22, 1.90)**	1.19	(.94, 1.50)
Having siblings	.15	(−.07, .38)	.92	(.66, 1.28)	**.72**	**(.55, .94)**
BMI	**-.04**	**(−.07, −.02)**	**1.06**	**(1.03, 1.11)**	1.05	(1.02, 1.08)
Family structure						
Single parent	**-.39**	**(−.58, −.20)**	**1.36**	**(1.04, 1.78)**	**1.40**	**(1.09, 1.79)**
Reconstituted	**-.31**	**(−.53, −.08)**	1.36	(.98, 1.89)	1.25	(.96, 1.63)

^a^*b* Unstandardized regression coefficient; reference category for age category = 16 yr. olds; for material affluence High; for parental status , both parents. Estimates in bold indicate statistical significance at the *p* < .05 level

In Table 3 the crude associations show that living in a single parent or in a reconstituted family structure was positively associated with hours/weekday screen-based passive entertainment, hours/weekday with other screen-based activities, and for those living in a reconstituted family with hours/weekday of gaming. In the adjusted model living in a single parent or in a reconstituted family structure remained positively associated with hours/weekday with other screen-based activities, and for those in reconstituted families also with hours/weekday of gaming. Other significant predictors of screen time activities were gender, age and BMI, the latter not for hours/weekday of gaming.

**Table 3:** Crude and adjusted associations of family structure with weekday screen-based passive entertainment, gaming, and other screen-based activities^a^

	Screen-based passive entertainment (hours/weekday)		Gaming (hours/weekday)		Other screen-based activities (hours/weekday)	
	b	95% CI	b	95% CI	b	95% CI
Crude						
Family structure						
Single parent	**.21**	**(.05, .38)**	.09	(−.09, .27)	**.36**	**(.16, .57)**
Reconstituted	**.23**	**(.04, .42)**	**.22**	**(.001, .43)**	**.63**	**(.39, .86)**
Adjusted						
Female sex	**-.29**	**(−.40, −.17)**	**-.84**	**(−.98, −.71)**	**.17**	**(.04, .31)**
Age category						
11 yrs. old	**-.46**	**(−.70, −.33)**	**-.06**	**(−.32, .20)**	**-1.37**	**(−1.60, −.13)**
13 yrs. old	**-.01**	**(−.22, .17)**	.23	(−.03, .48)	-.49	**(−.75, −.23)**
15 yrs. old	.20	(−.04, .32)	**.30**	**(.05, .55)**	-.06	(−.30, .19)
Material affluence						
Low	.05	(−.15, .24)	.02	(−.20, .23)	-.05	(−.28, .18)
Medium	.03	(−.13, .19)	-.05	(−.22, .12)	.03	(−.22, .16)
Having siblings	-.17	(−.38, .03)	.05	(−.16, .25)	.02	(−.21, .24)
BMI	**.04**	**(.01, .05)**	.02	(−.002, .05)	**.04**	**(.02, .07)**
Family structure						
Single parent	.15	(−.01, .31)	.09	(−.08, .26)	.25	(.06, .43)
Reconstituted	17	(−.01, .35)	**.26**	**(.05, .48)**	**.44**	**(.22, .66)**

^a^b=unstandardized regression coefficient; reference category for age category = 16 yr. olds; for material affluence = middle; for parental status = both parents. Estimates in bold indicate statistical significance at the *p* < .05 level

Results for screen time activities in weekends were largely in line with the ones reported for weekdays (not shown in Table 3). One notable exception was that the association between screen-based passive entertainment in weekends and reconstituted families remained significant in the adjusted analyses (b = .27; 95%CI: .08, .46, *p* = .005).

In Table 4, the crude associations demonstrate a significant relationship between living in a reconstituted family and total hours/weekday of screen time as well as exceeding two hours/weekday. There was also a positive but weaker association between living with a single parent and total hours/day of screen time. In the adjusted model, gender, age and BMI were significant predictors. Controlling for covariates had a small reduction on the strength of the observed associations.

**Table 4:** Crude and adjusted associations of family structure with total and recommended screen time^a^

	Total screen time (hours/weekday)		Screen time more than two hours per weekday	
	b	95% CI	Odds ratio	95% CI
Crude				
Family structure				
Single parent	**.68**	**(.23, 1.14)**	1.08	(.82, 1.43)
Reconstituted	**1.04**	**(.54, 1.54)**	**1.62**	**(1.14, 2.31)**
Adjusted				
Female sex	**-.96**	**(−1.27, −.66)**	**.65**	**(.54, .78)**
Age category				
11 yrs. old	**-1.91**	**(−2.51, −1.30)**	**.37**	**(.28, .50)**
13 yrs. old	-.34	(−.96, .28)	.81	(.59, 1.11)
15 yrs. old	.45	(−.11, 1.02)	1.11	(.80, 1.54)
Material affluence				
Low	.05	(−.48, .58)	.90	(.65, 1.26)
High	-.05	(−.48, .38)	.95	(.74, 1.23)
Having siblings	-.07	(−.57, .43)	.85	(.58, 1.25)
BMI	**.10**	**(.05, .16)**	**1.06**	**(1.02, 1.09)**
Family structure				
Single parent	**.50**	**(.08, .93)**	.99	(.74, 1.31)
Reconstituted	**.85**	**(.37, 1.33)**	**1.48**	**(1.02, 2.14)**

^a^b = unstandardized regression coefficient; reference category for age category = 16 yr. olds; for material affluence = middle; for parental status = both parents. Estimates in bold indicate statistical significance at the *p* < .05 level

Again, the results for total and recommended screen time in weekend days were largely in line with the one reported for weekdays (not shown in Table 4), although only borderline significant associations were found in the adjusted analyses for total screen time and single parent families (b = .43, 95%CI: −.05, .91, *p* = .08), and for recommended screen time and reconstituted families (OR = 1.89; 95%CI: .91, 3.93, *p* = .09).

For all but one, the interaction effects of family structure with sex, material affluence and having siblings were not statistically significant. A statistically significant interaction effect was found between family structure and material affluence for participation in organized sports only (χ^2^ [4] =13.9, *p* = .008). Living in a reconstituted family was associated with an increased risk for not participating in organized sports for young people who reported low (OR = 3.14, *p* = .004) and high material affluence (OR = 2.08, *p* = .01), but not those who reported medium material affluence (OR = .90, *p* = .57).

## Discussion

This study sought to examine if family structure was associated with young people’s MVPA, participation in organized sport and time spent on various screen activities after school. After adjusting for gender, age, material affluence, having siblings and BMI, the results showed that living in a single parent or a reconstituted family was negatively associated with days/week of 60 min MVPA and positively associated with hours/weekday with total screen time and hours/week with screen-based activities other than gaming and passive entertainment. Furthermore, living with a single parent increased the odds for less than 7 days/week of 60 min MVPA and for not participating in organized sport, while living in reconstituted families increased the odds for exceeding 2 h/weekday of total screen time and was positively associated with hours/day of gaming and with hours/weekend-days of screen-based passive entertainment.

### Family structure, physical activity and sport

One key finding was that living with a single parent or in a reconstituted family was negatively associated with number of days/week of 60 min MVPA, with the strongest relationship observed for single parent homes. No evidence was found that the association between MVPA and family structure differed across gender, material affluence and having siblings.

Youth from single parent families had also increased odds for less than 7 days/week of 60 min MVPA. We have not identified other studies that have examined and documented a relationship between family structure and the physical activity recommendations. This finding should therefore be of public health interest, as it suggests that Norwegian youth living with single parents might be potentially “at increased risk”, for not fulfilling the physical activity recommendations. We also found increased odds for not participating in organized sport when living with a single parent. Furthermore, there was an interesting interaction effect observed for young people living in a reconstituted family that had an increased risk for not participating in organized sports if they reported low or high material affluence. This finding suggests that complex processes are involved, and highlights the need for more in depth research on these relationships. Our results are largely in line with the findings from a study conducted in Canada [31], where young people from both single-parent and reconstituted families were less likely to participate in organized sport than those living with both their biological parents. In the Canadian study, perceived family wealth partially mediated the relationship between family structure and organized PA participation [32]. Family affluence was in our study a predictor of organized sport participation as well, suggesting that economic factors, such as costs related to sport participation, may partly explain the observed relationship.

In addition, it has been suggested that parents’ time provides the combination of support and control associated with positive child outcomes [24, 35]. Qualitative studies have reported that children in single parent families experience more barriers for engaging in physical activities than children in traditional families, and receive less parental support due to lack of free time, work load, and household responsibilities [35]. In Norway, the majority of sports organized for young people is carried out by volunteers among parents [68], i.e. parents are expected to be involved in the sports clubs doing voluntary work. Lack of time may thus influence the ability to take on such responsibilities. Young people will also often be dependent on their parents for transportation to both training facilities and to competitions. Another factor that has been reported to restrict engagement in physical activities among children in non-traditional families is time to travel to visit biological parents [35]. Thus, issues related to time constraints seem possible additional explanations for the identified relationships.

### Family structure and screen time use

A second key finding was that living with a single parent or in a reconstituted family was positively associated with hours/weekday of total screen time and with hours/day of screen-based activities other than gaming and passive entertainment, with the strongest associations found for children in reconstituted families. This type of family structure also increased the odds for exceeding 2 h/weekday of total screen time and was positively associated with hours/day of gaming. Adjusting for covariates had only a small reduction on the strength of the associations. No evidence was found that the association between screen time use and family structure differed across gender, material affluence and having siblings.

These results contrast the findings of McMillan et al. (2015) who based on the same screen time items, found generally no significant differences between Canadian youth from traditional and non-traditional families in hours/week of total screen time, nor higher odds to exceed screen time guidelines, or to be in the highest quartile of video game or computer use [21]. A study of English youth found higher levels of sedentary activities among those in single-parents but not in step-parent families [35]. Another English study found that boys, but not girls, had higher levels of total sedentary time on weekdays and weekend, and higher levels of weekday computer use and of weekend TV viewing, when living with a single parent compared with dual-parent households [37]. Several studies [6, 20, 21, 35, 37, 39, 69] have not found an association between family structure and TV viewing. Our results of screen-based passive entertainment (including watching TV) are largely in line with these findings with the exception of a significant and positive association between reconstituted families and screen-based passive entertainment in weekends.

The reasons for the observed relationships in the current study are likely multifaceted. Less involvement in physical activity and sport for these adolescents may be one factor that could result in sedentary pursuits [35]. A family level construct that has been linked to positive health behaviors is family cohesion, defined as the “emotional bonding that family members have toward one another” [70]. Some studies indicate that stepparents are less committed to their non-biological children [22, 71], it has also been suggested that reconstituted families need time to establish a new family structure and reorganize [72]. The stress and instability perspective suggests that changes in family structure may disrupt the equilibrium of family environments for many years, with elevated stress that interrupts effective parenting behaviors [73, 74]. These aspects, together with the time factor, may result in less support and involvement in parenting, as well as challenges when enforcing screen time limitations on youth that, in sum, influence screen-based behaviors. The findings in the current study suggest the need for a more in-depth assessment of the relationship between family structures and screen-based behaviors that can uncover the processes and shed light of the mechanisms through which family structures may influence the behaviors.

### Strengths and limitations

A strength of the study was the large sample of Norwegian youth with sufficient statistical power to examine main and interaction effects related to family structure. The study also controlled for several potential covariates. Another strength was the use of well-established measures developed by expert teams within the international HBSC study, a study that has comprehensive methodological data collection procedures. The study has several limitations. A known limitation is that all data were self-reported, which is known to have recall and reporting bias. However, most of the items have been documented to have satisfying validity and reliability [45].

Another limitation was that the proportion reporting other types of family structures was in the current study insufficient to examine the relationships with less common, but potentially important family types. We also lacked information on how long the participants had been living in their current family structure, limiting our ability to study the processes involved. Another limitation is the use of cross-sectional data, which does not allow for causal inference. In addition, the issue with omitted variable bias makes it difficult to propose any causality. The factors that affect the type of family structure parents choose may also affect parental resources, as well as the physical activity and sedentary behaviors of the children. If a larger set of parental factors, such as educational level, could have been included as covariates, more of the potential unobserved sources of the observed association could have been accounted for.

### Implications

The finding that family structure was a correlate for MVPA, sport participation and screen-time activities among Norwegian youth, should be of interest for public health researchers and practitioners as the proportion of young people living in family structures consisting of others than both their biological mother and father, is substantial. The study suggests that young people living with a single parent are “at risk” for not meeting the MVPA-recommendations and for not participating in organized sport. This is an important finding as sport can contribute to a significant amount of young peoples’ total physical activity, in particular vigorous activity [42, 43, 75]. In a Norwegian setting, it could be worthwhile to address structural and organizational barriers that single parents may experience. Those living in reconstituted families were “at-risk” for exceeding 2 h of screen-time use, with higher levels of both gaming and screen-based activities other than passive entertainment. So far, few studies have identified correlates of sedentary time with an attention to the after-school period, and those that exist have mainly focused on TV viewing [8, 44]. The fact that computer use has sharply increased [4], that it has been limited effect of interventions to reduce sedentary time [8], and that the relationship with many of the determinants of sedentary behavior remain inconsistent [76], underline the importance of more studies on correlates. Most of the research on family structure has typically assessed single versus dual families. The identified relationships between living in a reconstituted family and lower levels of physical activity as well as higher levels of screen time use compared to two parent families are interesting and warrant further investigation of the mechanisms involved. A recent nationally representative study of Norwegian 8 year olds found that objectively measured overweight was more prevalent among children of divorced parents [77]. The identified relationships between family structure and physical activity and sedentary activities could thus be a pathway to overweight. Longitudinal and also studies with a qualitative design could shed light on the processes and the complexity of the examined associations.

As the majority of the participants in this study did not meet the guidelines for PA and SB regardless of family structure, universal interventions are warranted to increase levels of PA and reduce levels of SB. However, the study findings suggest that family structure may be a factor to account for in the development of interventions, for example by examining ways to intensify interventions for single and reconstituted families (i.e. proportionate universalism [78]). It would also be of interest to examine whether family structure acts as a moderator for intervention effects.

## Conclusion

The study demonstrated that family structure was a correlate of physical activity, sport participation and screen time behaviors among Norwegian youth. These findings suggest that family structure could be an important factor to take into account in the development of interventions and programs. More research on this topic is warranted.

## Abbreviations

BMI: Body mass index
CI: Confidence interval
EU: European Union
FAS III: Family affluence scale III
FIML: Full information maximum likelihood
HBSC: Health Behaviour in School- aged Children
MVPA: Moderate to vigorous physical activity
OR: Odds ratio
PA: Physical activity
SD: Standard deviation
WHO: World Health Organization
